# Haplotype inference in general pedigrees with two sites

**DOI:** 10.1186/1753-6561-5-S2-S6

**Published:** 2011-05-28

**Authors:** Duong D Doan, Patricia A Evans

**Affiliations:** 1 Faculty of Computer Science University of New Brunswick, Fredericton, New Brunswick, Canada

## Abstract

**Background:**

Genetic disease studies investigate relationships between changes in chromosomes and genetic diseases. Single haplotypes provide useful information for these studies but extracting single haplotypes directly by biochemical methods is expensive. A computational method to infer haplotypes from genotype data is therefore important. We investigate the problem of computing the minimum number of recombination events for general pedigrees with two sites for all members.

**Results:**

We show that this NP-hard problem can be parametrically reduced to the Bipartization by Edge Removal problem and therefore can be solved by an *O*(2*^k^* · *n*^2^) exact algorithm, where *n* is the number of members and *k* is the number of recombination events.

**Conclusions:**

Our work can therefore be useful for genetic disease studies to track down how changes in haplotypes such as recombinations relate to genetic disease.

## Background

Human genomes contain two copies of each chromosome. Research shows that single chromosomes, called haplotypes, are useful to study complex genetic diseases [1]. While genomic data, called genotypes, are abundant and easy to collect, haplotypes are rare and much more difficult to obtain by a biochemical method. Therefore, a computational method to infer haplotypes from genotype data, called haplotyping, is necessary. Genotypes can be obtained from a population group where relationships between members are unknown or from a multigenerational family pedigree with known relationships between members. We only consider pedigree data in this paper.

**Figure 1:**
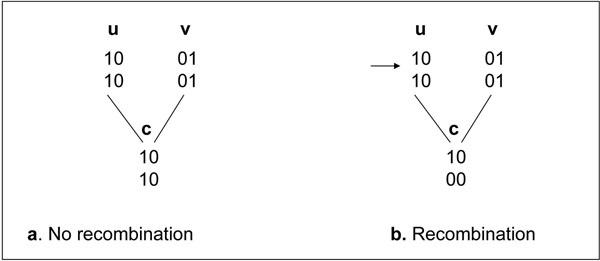
**Non-recombination vs. recombination.** Recombination happens between sites 1 and 2 of parent u and the child c receives a combined haplotype from parent u. Here haplotypes of members are displayed in columns.

In the absence of recombination events, haplotypes of members in a pedigree follow the Mendelian law of inheritance, where the two haplotypes of a child are transferred from its parents, one haplotype from its father and the other from its mother. Various haplotyping algorithms exist for non-recombinant pedigree data [2-5], especially a linear time algorithm for non-recombinant tree pedigrees [2] and a near-linear time algorithm for non-recombinant general pedigrees [3]. Haplotype inference is complicated by recombination events and the complex structures of the data themselves. Recombination happens when complementary parts of both of a parent’s haplotypes can be inherited as a single combined haplotype of a child (Figure 1). Structures of the pedigree data can be complex with loops, where there are multiple inheritance paths between some family members.

The haplotyping problem has been studied extensively in the last few years, both for pedigree and population data. If recombinations are allowed, the problem of inferring haplotypes for pedigrees with the minimum number of recombinations is NP-hard [6]. In fact, inferring haplotypes for pedigrees with minimum number of recombinations is NP-hard even for general pedigrees with only two sites or tree pedigrees with multiple sites [7]. For reconstructing haplotype configurations for pedigree data, Qian and Beckmann [8] proposed a rule-based algorithm with a time complexity *O*(2*^d^n*^2^*m*^3^), where d is the largest number of children in a family, *n* is the number of members and m is the number of sites. The main principle of their algorithm is that the best haplotype configuration for pedigree data is the one that minimizes the number of recombination events (the Minimum-Recombinant Haplotype Configuration (MRHC) problem). In [9][6] Li and Jiang proposed an *O*(*dmn*) block-extension algorithm for the MRHC problem using a greedy heuristic to resolve adjacent sites. However, as discussed in [10], this algorithm did not always find the haplotypes that minimized the number of recombinations, and worked under some restrictions. In order to improve the performance and handle missing data, an integer linear programming (ILP) formulation [10] was proposed, in which a branch-and-bound algorithm was used to narrow the search space. When the number of recombination events is strictly smaller than a positive number *k*, an *O*(*mn* · log*^k^*^+1^*n*) time probabilistic algorithm is given on tree pedigrees [11].

We study the minimum haplotype configuration for general pedigrees, where each member in a pedigree has only two sites; even this restricted problem is NP-hard [6]. We assume that there are no data missing and no data errors from the input genomic data. We prove that our problem can be reduced to the problem of finding the *line index* of a *signed graph*[12]. We further show that finding the line index of a signed graph can also be reduced to the Bipartization by Edge Removal problem. Our problem can therefore be solved by a fixed-parameter algorithm with a running time of *O*(2*^k^* · *n*^2^), where *n* is the number of members and *k* is the number of recombination events.

## Concepts

A member is an individual. A set of members is called a *family* if it includes only two parents and their children; it is a *parent-offspring trio* (hereafter a *trio*) if only two parents and one child are considered. A set of families connected through known family relationships is a *pedigree.* A parent is an *internal parent* if it is a child of another family; it is an *external parent* otherwise.

In diploid organisms, a cell contains two copies of each chromosome. The description data of the two copies are called a *genotype* while those of a single copy are called a *haplotype.* A specific location in a chromosome is called a *site* and its state is called an *allele.* There are two main types of sites, *microsatellites* and *single nucleotide polymorphisms.* A microsatellite site has several different states while a single nucleotide polymorphism (*SNP*) site has exactly two possible states, denoted by 0 and 1. Only SNPs with two possible states are considered in this paper, as in other works on haplotype inference. If the states at a specific site in two haplotypes are the same, then this site is a *homozygous* site (0-0 or 1-1); if they differ, it is *heterozygous* (0-1 or 1-0). Two haplotypes combine together to form one genotype. Each member *u* has two haplotypes, denoted by *h*1*_u_* and *h*2*_u_*, which are vectors of 0 and 1’s of length m, where m is the number of sites. The genotype of *u*, *g_u_*, is a vector of 0’s, 1’s and 2’s of length m, where *g_u_*[*i*] = 0 means *h*1*_u_*[*i*] = 0 = *h*2*_u_*[*i*], *g_u_*[*i*] = 1 means *h*1*_u_*[*i*] = 1 = *h*2*_u_*[*i*], and where *g_u_*[*i*] = 2 means {*h*1*_u_*[*i*], *h*2*_u_*[*i*]} = {0,1}. We say *h*1*_u_* and *h*2*_u_* are consistent with *g_u_.* The complement haplotype of a haplotype *h* at a heterozygous site is denoted by , where  so,  and .

The problem in this paper is to find the haplotypes *h*1*_u_* and *h*2*_u_* for all members *u* that minimize the number of recombination events, given their genotypes *g_u_.* A set of haplotypes found for all members is called a *haplotype configuration.* When *g_u_*[*i*] = 0 or 1, then *h*1*_u_*[*i*] and *h*2*_u_*[*i*] are known, but if *g_u_*[*i*] = 2, we may not yet know the value of *h*1*_u_*[*i*] and *h*2*_u_*[*i*], in which case we give them the value “?”, and say that the site is *unresolved.* Our problem is defined as follows.

**2-site-MRHC***_opt_***:***Given the genotypes of a general pedigree P containing n members, where each member has only two sites, find a haplotype configuration that minimizes the number of recombination events.*

This optimization problem, called 2-site-MRHC*_opt_*, was proven NP-hard [7]. We investigate the corresponding decision version of 2-site-MRHC*_opt_*.

**2-site-MRHC***_k_*: *Given a positive integer k and the genotypes of a general pedigree P containing n members*, *where each member has only two sites*, *is there a haplotype configuration with at most k recombination events explaining P?*

There is a correspondence between an optimization version and a decision version of the MRHC problem. We can get a result for the optimization version of the problem by trying parameter *k* with 0 and increasing its value step by step to solve the decision version until the problem answer is Yes. On the other hand, we can immediately get a result for the decision version of the problem from a result of the optimization version.

## Methods

We construct a pedigree graph to represent the 2-site-MRHC*_k_* problem.

### Label members

Given a member *u* and its two sites *i* and *j*, if sites *i* and *j* are both heterozygous or both homozygous, the member is *labeled*. If only one site is homozygous and the other site is heterozygous, the member is *unlabeled*.

If *i* and *j* are both homozygous with the same value (*g_u_*[*i*] = *g_u_*[*j*] = 0 or *g_u_*[*i*] = *g_u_*[*j*] = 1), *u* is labeled green. If i and j are both homozygous with different values (*g_u_*[*i*] = 0 and *g_u_*[*j*] = 1, or *g_u_*[*i*] = 1 and *g_u_*[*j*] = 0), u is labeled *red*. If i and j are both heterozygous, *g_u_*[*i*] = *g_u_*[*j*] = 2, u is labeled *grey*. A member is *resolved* if it is labeled red or green. A member is *unresolved* if it is labeled grey. A grey member u will later be resolved green if *h*1*_u_*[*i*] = *h*1*_u_*[*j*] = 0 or *h*1*_u_*[*i*] = *h*1*_u_*[*j*] = 1. It is resolved red otherwise. The resolution of a grey member depends on its adjacent members.

### Insert positive edges

**Figure 2:**
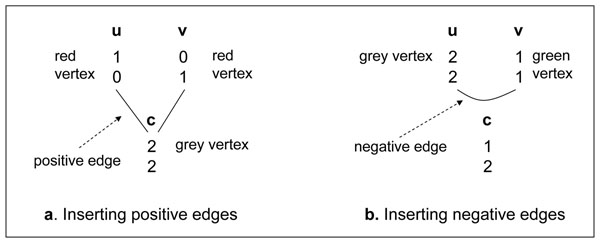
**Inserting positive and negative edges.** Here genotypes of members are displayed.

If *u* is a parent of v and both *u* and v are labeled, we insert a *positive edge*, *e_pos_*(*u*, *v*), between *u* and *v*. A positive edge *e_pos_*(*u*, *v*) means the label of *u* and the label of *v* should be the same once resolved, unless a recombination occurs in *u*. The reason for this is that if there is no recombination in *u*, then *v* receives one full haplotype from u and another full haplotype from another parent based on the Mendelian law of inheritance. Therefore, the label of *u* and the label of *v* should be the same if there is no recombination; otherwise, there is a recombination event in *u*. If *u* is a resolved vertex and there is a positive edge between *u* and a grey vertex *v*, we color *v* the same as the color of *u*, since a recombination event at *u* is not detectable and does not affect the color of *v*. For example, member *c* in Figure 2.a would be colored red because its parental member *u* is red, and *u* and *c* are linked by a positive edge.

### Insert negative edges

We also consider a trio with two parents, *u* and *v*, and a child *c*. If both parents are labeled but the child is not labeled, we insert a *negative edge*, *e_neg_*(*u*, *v*), between *u* and *v*. A negative edge *e_neg_*(*u*, *v*) means *u* and *v* should be resolved with different labels, unless there is a recombination event in one parent of *c*.

This phenomenon can be explained as follows. If there is no recombination and *u* and *v* have the same resolved label, i.e., both red or both green, then sites *i* and *j* of *c* must be both homozygous or both heterozygous based on the Mendelian law of inheritance. Because only one site of *c* is homozygous and the other site is heterozygous, one recombination occurs if *u* and *v* have the same label when resolved, but no recombination occurs if they are resolved differently.

Figure 2 shows positive and negative edges inserted between members in the pedigree.

### Process unlabeled members

So far, we have processed labeled members. Now we process an unlabeled member *u* that has one homozygous site and one heterozygous site.

If *u* is a child in its previous generation, a negative edge is inserted between two parents of *u* as discussed in Subsection . If *u* is a parent of a child *c*, there is no way to detect whether there is a recombination event in *u* caused by haplotype shuffling or not. This fact can be explained as follows. Without loss of generality, suppose *g_u_*[*i*] = 0 and *g_u_*[*j*] = 2, the haplotype pair of *u* inferred would be *h*1*_u_* = 01 and *h*2*_u_* = 00. The possible mixed haplotypes transferred to *c* from *u* are still either {01} or {00}. In both cases, we can explain *u* as a member with no recombination event by pointing the haplotype of *c* that is received from *u* to the appropriate haplotype of *u*.

Because we use unlabeled child members to insert negative edges only and there is no way detect haplotype shuffling in unlabeled parental members, we only consider members that are labeled from now on. Once labeled members are resolved, we can resolve unlabeled members accordingly.

### Pedigree graph

Pedigree *P* can be considered to be an undirected graph *G* = (*V*, *E*)*.* Each vertex *v* ∈ *V* is a member with three possible labels, red, green, and grey. Each edge *e*(*u*, *v*) ∈ *E* is either a positive edge, *e* ∈ *E_pos_*, or a negative edge; *e* ∈ *E_neg_*, (*E* = *E_pos_* ∪ *E_neg_*)*.* Graph *G*, set up this way, is a signed graph [12]. Let *N*(*u*) be the set of adjacent vertices of *u.* Let *w*(*e*) be the weight of edge *e*. If *e* is a positive edge, *w*(*e*) = +1 . If *e* is a negative edge, *w*(*e*) = –1.

**Observation 1.***There are at most n vertices and O(n) edges in the pedigree graph.*

There are *n* members in the pedigree. A vertex is created for each member, except for unlabeled members with one site homozygous and one site heterozygous. Thus there are at most *n* vertices in the pedigree graph.

Except for external parents, a member has two positive edges linking it to two parents. Therefore, the number of edges in the graph is linear in the number of child members. If a member is an unlabeled member, the two positive edges linking two parents and the child are replaced by a negative edge between the two parents. Thus the number of edges in the pedigree graph is *O*(*n*)*.*

The 2-site-MRHC*_k_* problem can now be solved by determining if we can label every grey vertex in *G* either red or green such that if we partition the set of vertices *V* into (*V_red_*, *V_green_*) and let E* be the set of edges between *V_red_* and *V_green_* then(1)

Given a pedigree graph, any two adjacent members linked by a positive edge should be in the same partition, and any two adjacent members linked by a negative edge should be in different partitions. Any edge whose constraint is not satisfied represents a recombination event between the two adjacent members, or, in the case of a negative edge having endpoints in the same partition, between one parent and the child. Equation 1 thus counts the number of recombination events in the whole pedigree and ensures that it is at most *k.* This problem can be reduced to the problem of finding the line index of a signed graph [12].

### Signed graph

A graph *G* = (*V*, *E*) is a *signed graph* if it has both positive and negative edges (*E* = *E_pos_* ∪ *E_neg_*) [12], where *w*(*e_pos_*) = 1 and *w*(*e_neg_*) = –1. Let (*V*_1_, *V*_2_) be a partition of *V*, and E* be the set of edges between *V*_1_ and *V*_2_. The *line index* of the cut (*V*_1_, *V*_2_) is defined as:(2)

The line index of graph G is defined as:(3)

The corresponding decision version of finding the line index of graph *G* is defined as follows.

**LineIndex***_k_*: *Given a signed graph G and a positive integer k*, *is there a line index of G at most k?*

Clearly, the 2-site-MRHC*_k_* problem can be reduced to the LineIndex*_k_* problem. We will show that the LineIndex*_k_* problem can be reduced to the Bipartization by Edge Removal problem, a classic NP-complete problem that is fixed-parameter tractable.

## Data deduction rules

Given the signed graph *G* constructed as above and a parameter *k* (*k* ≥ 0), we apply some data deduction rules to transform (*G*, *k*) to (*G*′, *k*′), where *G*′ is smaller than *G* and *k*′ ≤ *k.*

Let *G* \ *u* be the graph obtained from *G* by deleting the vertex *u* and all edges incident on *u.* Let *G* \ *e*(*u*, *v*) be the graph obtained from *G* by deleting edge *e*(*u*, *v*)*.* Let *N*(*u*) be the set of all adjacent vertices of *u.* Let  and  be the sets of all red vertices adjacent to *u* by positive edges and negative edges, respectively. Let  and  be the sets of all green vertices adjacent to *u* by positive edges and negative edges, respectively.

**Observation 1***If* ∑ *w*(*e_pos_*(*red*, *green*)) + ∑ *|w*(*e_neg_*(*green*, *green*)) + *w*(*e_neg_*(*red*, *red*)) *|*>*k*, *then G is a No-instance for 2-site-MRHC_k_.*

This observation is true based on Equation 1.

**Observation 2***If x is a grey vertex and**then x is labeled green. If x is a grey vertex and**then x is labeled red.*

If we label *x* oppositely (from red to green and vice versa), there are more than *k* recombination events in *G*.

**Observation 3***If x is a grey vertex and**then x is labeled green. If x is a grey vertex and**then x is labeled red.*

If  and *x* is labeled red then more than half of its incident edges cause recombination events. Thus *x* is labeled green. Similarly for the case .

**Reduction rule 1*** If a vertex u has degree 0*, *then* (*G*, *k*) *is a Yes-instance of 2-site-MRHC_k_ if and only if* (*G \ u*, *k*) *is a Yes-instance.*

**Proof 1*** This is true based on Equation 1. If u is a grey vertex*, *we arbitrarily relabel u to either red or green and put u in the corresponding partition. In both cases*, *k remains the same.*

**Reduction rule 2 ***If a grey vertex u has degree 1*, *then* (*G*, *k*) *is a Yes-instance of 2-site-MRHC_k_ if and only if* (*G \ u*, *k*) *is a Yes-instance.*

**Proof 2*** Let v be the vertex incident to u. If e*(*u*, *v*) *is a positive edge*, *u is labeled the same as the label of v and is put in the same partition as v. If e*(*u*, *v*) *is a negative edge*, *u is labeled oppositely from the label of v and is put in the different partition from the partition of v. In the both cases k remains the same.*

**Reduction rule 3*** If two vertices u and v are resolved with the same label* (*both red or both green*) *and there is an edge e_neg_*(*u*, *v*), *then* (*G*, *k*) *is a Yes-instance of 2-site-MRHC_k_ if and only if* (*G \ e_neg_*(*u*, *v*),*k* – 1) *is a Yes-instance.*

**Proof 3** T*his is true based on Equation 1.*

**Reduction rule 4*** If two vertices u and v are resolved with the same label and there is an edge e_pos_*(*u*, *v*), *then* (*G*, *k*) *is a Yes-instance of 2-site-MRHC_k_ if and only if* (*G \ e_pos_*(*u*, *v*), *k*) *is a Yes-instance.*

**Proof 4*** This is true based on Equation 1.*

**Reduction rule 5 ***If two vertices u and v are resolved with opposite labels and there is an edge e_pos_*(*u*, *v*), *then* (*G*, *k*) *is a Yes-instance of 2-site-MRHC_k_ if and only if* (*G \ e_pos_*(*u*, *v*), *k* – 1) *is a Yes-instance.*

**Proof 5*** This is true based on Equation 1.*

**Reduction rule 6*** If two vertices u and v are resolved with opposite labels and there is an edge e_neg_*(*u*, *v*), *then* (*G*, *k*) *is a Yes-instance of 2-site-MRHC_k_ if and only if* (*G \ e_neg_*(*u*, *v*),*k*) *is a Yes-instance.*

**Proof 6 ***This is true based on Equation 1.*

**Reduction rule 7*** If a grey vertex x has degree 2 with adjacent vertices u and v*, *three cases arise:*

1. *e*(*u*, *x*) and *e*(*x*, *v*) are both positive edges

(a) If *u* and *v* are both resolved with the same label (both red or both green), then (*G*, *k*) is a Yes-instance of 2-site-MRHC*_k_* if and only if (*G* \ *x*, *k*) is a Yes-instance.

**Proof 7*** We label x with the same label as the label of u and v and put x in the same partition as u and v.*

(b) If *u* and *v* are both resolved with opposite labels (one red and one green), then (*G*, *k*) is a Yes-instance of 2-site-MRHC*_k_* if and only if (*G* \ *x*, *k* – 1) is a Yes-instance.

**Proof 8 ***We label x with the same label as the label of either u or v. In both cases there is a recombination event in x or either u or v. Without loss of generality*, *if we label x with the same label as the label of u and put x in the same partition as u*, *then x and v are labeled oppositely. The positive edge between x and v cause a recombination event. By deleting x*, *the parameter k is reduced by 1.*

(c) If only *u* is resolved and *v* is not resolved, let *G*′ be the graph obtained from *G* by merging *u* and *x*, then (*G*, *k*) is a Yes-instance of 2-site-MRHC*_k_* if and only if (*G*′, *k*) is a Yes-instance.

**Proof 9*** We label x with the same label as the label of u and put x in the same partition as u. By merging u and x*, *edge e_pos_*(*x*, *v*) *becomes e_pos_*(*u*, *v*)*. Thus parameter k remains the same.*

(d) If neither *u* nor *v* resolved, let *G*′ be the graph obtained from *G* by merging *u* and *x*, then (*G*, *k*) is a Yes-instance of 2-site-MRHC_*k*_ if and only if (*G*′, *k*) is a Yes-instance.

**Proof 10 ***Even though neither u nor v is resolved*, *we can assume that x will be labeled the same as the future label of either u or v*, *say u. Be merging u and x*, *edge e_pos_*(*x*, *v*) *becomes e_pos_*(*u*, *v*)*. Thus parameter k remains the same.*

2. *e*(*u*, *x*) and *e*(*x*, *v*) are both negative edges

(a) If *u* and *v* are both resolved with the same label, then (*G*, *k*) is a Yes-instance of 2-site-MRHC*_k_* if and only if (*G* \ *x*, *k*) is a Yes-instance.

**Proof 11*** We label x with the opposite label from the label of u and v and put x in the different partition from u and v. Thus parameter k remains the same.*

(b) If *u* and *v* are both resolved with opposite labels, then (*G*, *k*) is a Yes-instance of 2-site-MRHC*_k_* if and only if ((*G* \ *x*), *k* – 1) is a Yes-instance.

**Proof 12** We *label x with a opposite label from the label of u. Therefore x and v have the same label and e_neg_*(*x*, *v*) *causes a recombination event. Once deleting x*, *edge e_neg_*(*x*, *v*) *is also removed and thus the parameter k is reduced by 1.*

(c) If only *u or v* resolved, say *u*, we transform *G* to *G*′ by labeling *x* with the opposite label of the label of *u* and deleting *e_neg_*(*u*, *x*)*.* (*G*, *k*) is a Yes-instance of 2-site-MRHC*_k_* if and only if (*G*′, *k*) is a Yes-instance.

**Proof 13*** We put x in a different partition from the partition of u. Since edge e_neg_*(*u*, *x*) *does not cause a recombination event*, *parameter k remains the same.*

(d) If neither *u* nor *v* resolved, then (*G*, *k*) is a Yes-instance of 2-site-MRHC*_k_* if and only if ((*G* \ *x*) ∪ *e_pos_*(*u*, *v*), *k*) is a Yes-instance.

**Proof 14 ***We label x with the opposite label from the label of u and v. If u and v are labeled the same in G*′ *then e_pos_*(*u*, *v*) *does not cause a recombination event and parameter k remains the same for graph G*′*. In this case x is labeled with opposite color from the color of u and v in G. If u and v are labeled oppositely in G*′ *then e_pos_*(*u*, *v*) *causes a recombination event. This corresponds to one recombination event in x or either u or v because whatever the color of x is* (*red or green*), *e_neg_*(*u*, *x*) *and e_neg_*(*x*, *v*) *cause a recombination event in G. Graph G*′ *will have the same parameter k.*

3. Either *e*(*u*, *x*) or *e*(*x*, *v*) is a positive edge. Without loss of generality, assume *e*(*u*, *x*) is a positive edge and *e*(*x*, *v*) is a negative edge.

(a) If *u* and *v* are both resolved with the same label, then (*G*, *k*) is a Yes-instance of 2-site-MRHC*_k_* if and only if (*G* \ *x*, *k* – 1) is a Yes-instance.

**Proof 15*** If we label x either red or green then either e_pos_*(*u*, *x*) *or e_neg_*(*x*, *v*) *causes a recombination event. Transforming graph G by deleting x*, *we obtain graph G*′ *with k*′ = *k* – 1.

(b) If *u* and *v* are both resolved with opposite labels, then (*G*, *k*) is a Yes-instance of 2-site-MRHC*_k_* if and only if ((*G* \ *x*), *k*) is a Yes-instance.

**Proof 16*** We label x with the same label as the label of u and put x in the same partition as u; e_neg_*(*x*, *v*) *does not cause a recombination event. Transforming graph G by deleting x*, *we obtain graph G*′ *with k*′ = *k.*

(c) If only *u or v* resolved

i. If *u* is resolved, we transform *G* to *G*′ by labeling *x* with the same label as the label of *u* and merging *u* and *x*, (*G*, *k*) is a Yes-instance of 2-site-MRHC*_k_* if and only if (*G*′, *k*) is a Yes-instance.

**Proof 17 ***If we label x the same as the label of u*, *e_pos_*(*u*, *x*) *does not cause a recombination event. Once merging u and x*, *edge e_neg_*(*x*, *v*) *becomes e_neg_*(*u*, *v*) *and parameter k remains the same.*

ii. If *v* is resolved, we transform *G* to *G*′ by labeling *x* oppositely from the label of *v* and deleting *e*(*x*, *v*), (*G*, *k*) is a Yes-instance of 2-site-MRHC*_k_* if and only if (*G*′, *k*) is a Yes-instance.

**Proof 18*** Similar to the previous proof.*

(d) If neither *u* nor *v* resolved, we transform *G* to *G*′ by merging *x* with *u.* (*G*, *k*) is a Yes-instance of 2-site-MRHC*_k_* if and only if (*G*′, k) is a Yes-instance.

**Proof 19 ***Even though neither u nor v resolved*, *we can assume that we will label x with the same label as the future resolved label of u. Thus e_pos_*(*u*, *x*) *does not cause a recombination event. Transforming graph G by merging x and u*, *edge e_neg_*(*x*, *v*) *becomes e_neg_*(*u*, *v*) *and we obtain graph G*′ *with k*′ = *k.*

There is no grey vertex with degree less than three in the graph once these data reduction rules are applied. Vertices with high degrees will likely be eliminated. Therefore, our data reduction rules will be very useful for various types of pedigrees, such as pedigrees containing many members with no children, pedigrees with small families, or pedigrees with very big families.

We performed experiments with the data deduction rules to see how efficient these rules are to reduce the sizes of pedigree graphs. We generated 20 random and highly complex pedigrees with many cycles based on the method presented in. Each member can have many spouses and some of its spouses can be its children or grandchildren. These pedigree structures may not be common for human but would be easily found in other species such as goats, fish, and horses. The numbers of members in families vary from 1000 to 10000; each member has two sites. From these pedigree structures and their genotype data, we constructed initial pedigree graphs with the numbers of vertices varying from 496 to 5021, positive edges from 350 to 3991, and negative edges from 90 to 936. Table 1 reports the numbers of vertices and edges in initial graphs and reduced graphs after the data deduction rules are used. The experiment shows that our data deduction rules can eliminate on average 99.5 % of vertices, and 99.6% of positive and negative edges in pedigree graphs. The data deduction rule program and test data are available at http://www.cs.unb.ca/profs/pevans/research/dr.

**Table 1:** Pedigree graphs before and after data deduction rules are used.

# of members	# of vertices in initial graph	# of positive edges in initial graph	# of negative edges in initial graph	# of vertices in reduced graph	# of positive edges in reduced graph	# of negative edges in reduced graph
1000	496	350	90	5	1	3
1500	769	509	100	6	5	1
2000	1022	685	186	5	2	2
2500	1248	771	167	6	5	1
3000	1553	1049	202	6	5	1
3500	1752	1230	268	18	11	5
4000	1991	1395	339	7	3	3
4500	2216	1655	415	7	4	2
5000	2535	2006	440	10	4	5
5500	2815	2371	556	11	9	1
6000	2037	1953	451	17	11	5
6500	3242	2142	484	14	11	2
7000	3486	2199	484	10	3	5
7500	3662	2668	661	16	9	5
8000	4972	3052	764	5	1	3
8500	4143	2464	506	7	7	1
9000	4444	3088	781	20	10	9
9500	4735	3365	867	14	6	6
10000	5021	3991	936	18	8	9

## Fixed-parameter algorithm

A NP-hard problem cannot be solved by a polynomial time algorithm unless P=NP. However, if we can restrict some parameters of the problem to small values, the running time of an algorithm for the problem can potentially be greatly reduced [13,14]. In this case, the problem is a parameterized problem and an algorithm that can solve the parameterized problem efficiently is a *fixed-parameter algorithm*. Formal definitions of parameterized problem and fixed-parameter algorithm [14] are as follows.

**Definition 1***A parameterized problem is a language L* ⊆ Σ* × Σ*, *where* Σ *is a finite alphabet. The second component is called the parameter of the problem.*

Practically, the parameter is a nonnegative integer or a set of nonnegative integers and therefore *L* ⊆ Σ* × ℕ. For (*x*, *k*) ∈ *L*, the size of the input is *n* = |(*x*, *k*)*|*, and the parameter is *k.*

**Definition 2***A parameterized problem L is a fixed-parameter tractable if it can be determined in f*(*k*) · *n^O^*^(1)^*time whether or not* (*x*, *k*) ∈ *L*, *where f is a computable function only depending on k. The corresponding class of problems is called FPT.*

A comprehensive survey of FPT problems can be found in [13] and [14].

### Transforming to bipartization by edge removal problem

We review an important property of a signed graph given by [12].

**Theorem 1***Let G be a signed graph. If we replace each edge with weight w*(*e*) > 0 *by two consecutive edges with weight -w*(*e*) *to get a graph G*′ *then l*(*G*) = *l*(*G*′)*.*

**Proof 20***Suppose* (*V*_1_, *V*_2_) *is a cut of G such that l*(*V*_1_, *V*_2_) = *l*(*G*)*. We replace each positive edge e*(*u*, *v*) *by two consecutive negative edges e*(*u*, *y*) *and e*(*y*, *v*), *where w*(*e*(*u*, *y*)) = *w*(*e*(*y*, *v*)) = –*w*(*e*(*u*, *v*)) *and y is a new vertex adjacent only to u and v. If u and v belong to the same partition we put y in a different partition from the partition of u and v. If u and v belong to different partitions*, *we arbitrarily put y in the same partition of either the partition of u or v. In all of the cases above we find the corresponding cut of G*′, *such that**. Therefore l*(*G*′) ≤ *l*(*G*)*.*

*Conversely*, *if**and y is a new vertex*, *then at least one edge incident to y is in the cut. We can find a corresponding cut of G*, (*V*_1_, *V*_2_) *such that**. Therefore l*(*G*′) ≥ *l*(*G*)*. Taken together*, *we get l*(*G*′) = *l*(*G*)*.*

Based on this property, the pedigree graph is transformed into a new graph by replacing every positive edge by two consecutive negative edges and adding new intermediate vertices. We obtain a new weighted graph *G*′ with all negative weighted edges. The graph *G*′ still has only *O*(*n*) vertices and *O*(*n*) edges. Equation 1 becomes(4)

This equation is to ensure that the total number of edges within *V*_1_ and edges within *V*_2_ is at most *k.* These edges once removed will make the graph bipartite.

To make the GBER algorithm [15] works on our partially colored graph, we merge all red vertices into one red vertex and all green vertices into one green vertex. We relabel the merged red vertex and the merged green vertex into two grey vertices, and insert *k* + 1 negative edges between them. We further transform our negative graph into a new graph with all positive edges by multiplying the weight of every edge by -1. Our problem becomes the Bipartization by Edge Removal problem [15,16]. The *k*-Bipartization by Edge Removal problem is defined as follows.

**Definition 3***Given a graph G* = (*V*, *E*) *and a positive integer k*, *is there a set C* ⊆ *E with |C|* ≤ *k whose removal produces a bipartite graph?*

Bipartization by Edge Removal is a classical NP-hard problem and is in FPT [15,16]. Its parametric dual is Max-Cut [17].

### FPT Algorithm for bipartization by edge removal

One efficient technique to tackle an FPT problem is *iterative compression.* It is first proposed by [16] in a breakthrough paper and has been shown to very useful for solving different minimization problems. The idea is that, given a solution of size (*k* + 1), we find a fixed-parameter algorithm that either constructs a solution of size *k* if one exists or outputs No if no solution exists. We iteratively compress the problem by reducing the size of its solutions step by step. Assuming the running time of the FPT algorithm is *O*(*f*(*k*) · *n^O^*^(1)^), the overall running time will be *O*(*n* · *f*(*k*) · *n^O^*^(1)^)*.*

Iterative compression technique is used by Guo et al. [15] to solve the Bipartization by Edge Removal problem with a running time of *O*(2*^k^* · *m*^2^), where *k* is the number of edges to be deleted to make the graph bipartite. The main idea of the algorithm is as follows.

Given a graph *G* = (*V*, *E*) where *E* = {*e*_1_, …, *e*_m_}. Let *G_i_* be a graph induced by edges {*e*_1_, …, *e_i_*} of *G* (1 ≤ *i* ≤ *m*). If *i* = 1, the optimal edge bipartization set of *G*_1_ is empty. If i > 1, let *X* be an optimal edge bipartization set of *G_i_* = *G*[*e*_1_, …, *e_i_*] and |*X*| = *k*′*.* Consider graph *G_i_*_+1_ = *G*[*e*_1_, …, *e_i_*_+1_]. If *X* is not an optimal edge bipartization set for *G_i_*_+1_ then *X*′ = *X* ∪ {*e_i_*_+1_} is clearly an optimal edge bipartization set for *G_i_*_+1_. From the edge bipartization set *X*′ of size *k*′ + 1, we find an edge bipartization of size at most *k*′ or show that no such edge bipartization of size at most *k*′ exists. The algorithm assumes that an edges bipartization *Y* which is smaller than *X*′ must be disjoint from *X*′, *Y* ∩ *X*′ = ø. This assumption can be made without loss of generality by a simple graph transformation. We replace each edge in *X*′ by three consecutive edges and choose the middle edge to be in the new *X*′*.* This graph transformation preserves the parities of lengths of all cycles. Therefore the transformed graph has an edge bipartization set of size *k*′ if an only if the original graph has an edge bipartization set of size *k*′*.* Let mapping Φ: *V*(*X*′) → {*A*, *B*} be a valid partition of *V*(*X*′) if for each {*y*, *z*} ∈ *X*, we have Φ(*y*) ≠ Φ(*z*). Let *A*_Φ_ be Φ^–1^(*A*) and *B*_Φ_ be Φ^–1^(*B*)*.* We enumerate all 2*^k^*′ valid partitions Φ of *V*(*X*′)*.* For each valid partition Φ we find a minimum edge cut *Y* in *G\X*′ between *A*_Φ_ and *B*_Φ_*.* In other words, we use *X*′ to partially color *G* and from the partially colored graph we compute a smaller bipartization set *Y.* This compression step is the core of the algorithm. We have the following theorem from [15].

**Theorem 2***Consider a graph G* = (*V*, *E*) *and a minimal edge bipartization set X*′ *for G. For a set of edges Y* ⊆ *E with X*′ ∩ *Y* = ø, *the followings are equivalent:*

(*1*) *Y is an edge bipartization set for G.*

(*2*) *There is a valid partition* Φ *for V*(*X*′) *such that Y is an edge cut in G\X*′ *between A*_Φ_=Φ^–1^(*A*) *and B*_Φ_=Φ^–1^(*B*)*.*

**Figure 3:**
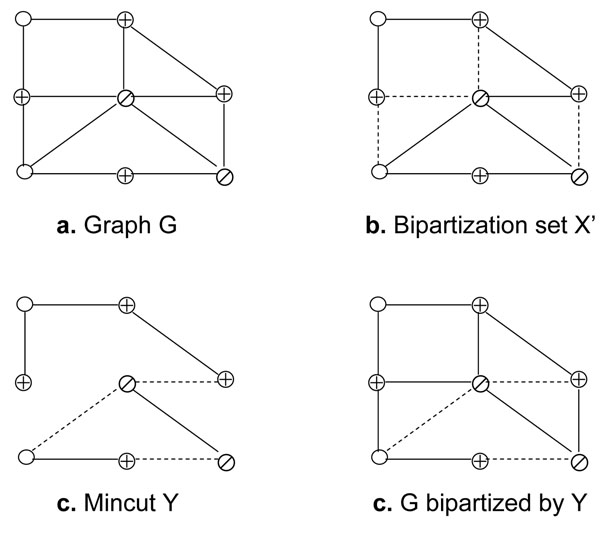
**Compression step.** The edge bipartization set is compressed by finding a mincut.

Consider a graph *G* in Figure 3.a where ⊕ denotes a red vertex, ⊘ denotes a green vertex, and O denote a grey vertex. A minimal edge bipartization set *X*′ illustrated by dashed lines is given in Figure 3.b. We compute a mincut *Y* for *G\X*′ as in Figure 3.c and *Y* is the minimum mincut for *G* in Figure 3.d.

**Theorem 3***The 2-site-MRHC_k_ problem is solvable in O*(*2^k^* · *n*^2^) *time.*

**Proof 21***Setting up the pedigree graph takes O*(|*V*|) *time. Transforming the pedigree graph into a graph with all negative edges takes O*(*|E|*) *time and transforming the negative graph into a graph with all positive edges takes O*(*|E|*) *time. The Bipartization by Edge Removal problem can be solved in O*(2*^k^* · *m*^2^). *Our graph is sparse with the number of edges linear in the number of vertices*, *so the overall running time of our algorithm is O*(2*^k^* · *n*^2^)*.*

### Extensions to pedigrees with more than two sites

**Figure 4:**
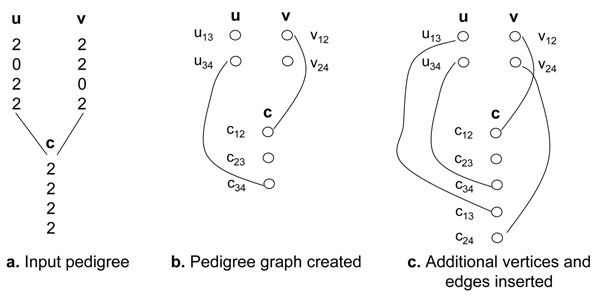
**Graphs from a pedigree with multiple sites.** Additional vertices are needed in order to capture the relationships between multiple pairs of sites in adjacent members of the pedigree.

Our method can be extended to work with pedigrees with more than two sites. In order to detect a recombination event in a member, it is necessary to have at least two heterozygous sites; one on each side of the recombination breakpoint. For example, we cannot detect a recombination between sites 1 and 2 of member u in Figure 2.a because the two haplotypes of u would be the same. However, we may able to detect a recombination between sites 1 and 3 of member u in Figure 4.a by comparing its different haplotype versions. We will capture constraints between pairs of closest heterozygous sites and pairs of closest homozygous sites between members and use them to detect recombination events. We create a grey vertex between two closest homozygous sites, and a red or green vertex between two closest homozygous sites of a members, depending on their genotype data. A vertex *u_ij_* is created between site *i* and site *j* of a member *u*. For example, we create a grey vertex *u*_13_ between site 1 and site 3, and a grey vertex *u*_34_ between site 3 and site 4 of member *u*. We insert a positive edge between a parental vertex *u_ij_* and a child vertex *c_ij_*. For example, we insert a positive edge between vertex *u*_34_ and vertex *c*_34_ in Figure 4.a. We insert a negative edge between two parental vertices *u_ij_* and *v_ij_* if there is not a vertex between sites *i* and *j* of their common child *c*.

The main difference between a pedigree with two sites and a pedigree with multiple sites is that besides vertices and edges created between closest heterozygous sites and closest homozygous sites, we may need to create additional vertices and edges for pedigrees with multiple sites to capture all constraints in a pedigree. For example, Figure 4.c shows an additional vertex *c*_13_ is created in *c* and a positive edge is inserted between vertices *u*_13_ and *c*_13_ to capture constraint between sites 1 and 3 of *u* and *c*. The number of total vertices in a member including additional vertices can be *O*(*m*^2^), where *m* is the number of sites in a members.

Additional vertices and edges are created in a member by the need of its adjacent members. They actually represent overlapped information. For example, vertex *c*_13_ can be represented by vertices *c*_12_ and *c*_23_. Thus when we solve the pedigree graph, we have to ensure that vertices are resolved consistently. For example, if vertices *c*_12_ and *c*_23_ are later resolved green and vertex *c*_13_ is resolved red, there is a parity conflict. The reason is that *h*1*_c_*[1] = *h*1*_c_*[2] = *h*1*_c_*[3] for green vertices *c*_12_ and *c*_23_. However, *h*1*_c_*[1] ≠ *h*1*_c_*[3] for red vertex *c*_13_. Therefore, a fixed-parameter algorithm for general pedigrees with multiple sites needs to ensure information consistency. We will investigate this problem in our future work.

## Conclusion

We have shown that the MRHC problem for general pedigrees with two sites can be reduced to the line index of a signed graph, and the line index of a signed graph can, in turn, be reduced to the Bipartization by Edge Removal problem. Therefore we can solve the MRHC problem for general pedigrees with two sites with an *O*(2*^k^* · *n*^2^) fixed-parameter algorithm. Future work will extend the current method to deal with genetic data with more than two sites.

## Competing interests

The authors declare that they have no competing interests.

## Authors’ contributions

DDD designed the algorithm and drafted the manuscript. PAE supervised the research, assisted in crafting the algorithm and polished the manuscript. Both authors read and approved the final manuscript.
